# Supplementary material for: Parasitism Features of a Fig Wasp of Genus Apocrypta (Pteromalidae: Pteromalinae) Associated with a Host Belonging to Ficus Subgenus Ficus
Source: Insects. 2023 May 3;14(5):437. doi: 10.3390/insects14050437 (PMC10231072; doi:10.3390/insects14050437)
Supplement: Supplementary file 1 [file insects-14-00437-s001.zip › insects-2343718-supplementary.pdf]

Parasitism features of a fig wasp of genus *Apocrypta* (Pteromalidae: Sycoryctinae) associated with a host belonging to *Ficus* subgenus *Ficus*

PO-AN CHOU[1], ANTHONY BAIN[2,3], BHANUMAS CHANTARASUWAN[4] and HSY-YU TZENG[1*]

[1]Department of Forestry, National Chung Hsing University, Taichung 402, Taiwan.

[2]Department of Biological Science, National Sun Yat-sen University, Kaohsiung 804, Taiwan.

[3]International PhD Program for Science, National Sun Yat-sen University, Kaohsiung 80424, Taiwan.

[4]Thailand Natural History Museum, National Science Museum, Pathum Thani, Thailand.

Correspondence: Hsy-Yu Tzeng, Fax: +86 04 2287 3628; E-mail: erecta@nchu.edu.tw

Table S1 Sampling information of the species belonged to *Ficus* subgenus *Sycomorus* selected in this study.

***Ficus* spp.**	***F. fistulosa***	***F. hipida***	***F. semicordata***	***F. variegata* var. *chlorocarpa***
Location	Dinghusan, Guangdong, China	Dinghusan, Guangdong, China	Phuchifa, Chiang Lai, Thailand	Shimen, Guangdong, China
Date	July 20th 2018	July 20th 2018	November 26th 2018	August 17th 2017
Fig wasp community	*Ceratosolen constrictus**	*C. solmsi**	*C. gravelyi**	*C. appendiculatus**
	Sycophaga sp.1	*A. bakeri*	*Sycophaga* sp.2	*Sycophaga spinitarsus*
	Apocrypta varicolor	*P. pilosa*	*Apocrypta* sp.1	*A. caudata*
	Philotrypesis sp.1	*Philotrypesis* sp.2	*Philotrypesis* sp.3	*P. bimaculata*
			Sycoryctes trifemmensis	*Sycoryctes patellaris*

Asterisks represent pollinator species. Other wasps are non-pollinating fig wasp (NPFW). Note the *Apocrypta* sp.1 associated with *F. semicordata* is a different species from the studied *Apocrypta* sp. associated with *F. pedunculosa* var. *mearnsii*.

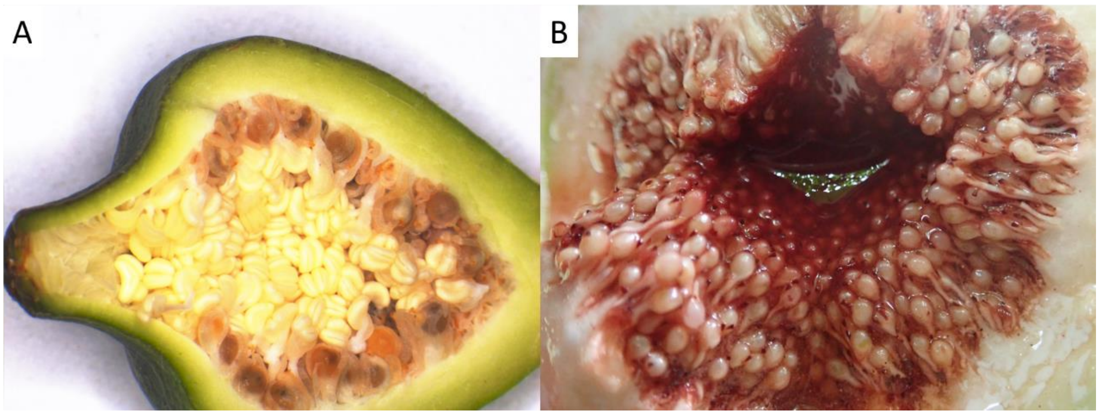

Figure S1 Difference between the cavities of male figs in subgenus *Ficus* and subgenus *Sycomorus*. (A) *F. pedunculosa* var. *mearnsii*, abundant male flowers cram the cavity. (B) *F. auriculate*, a lumen space exists with fluid.

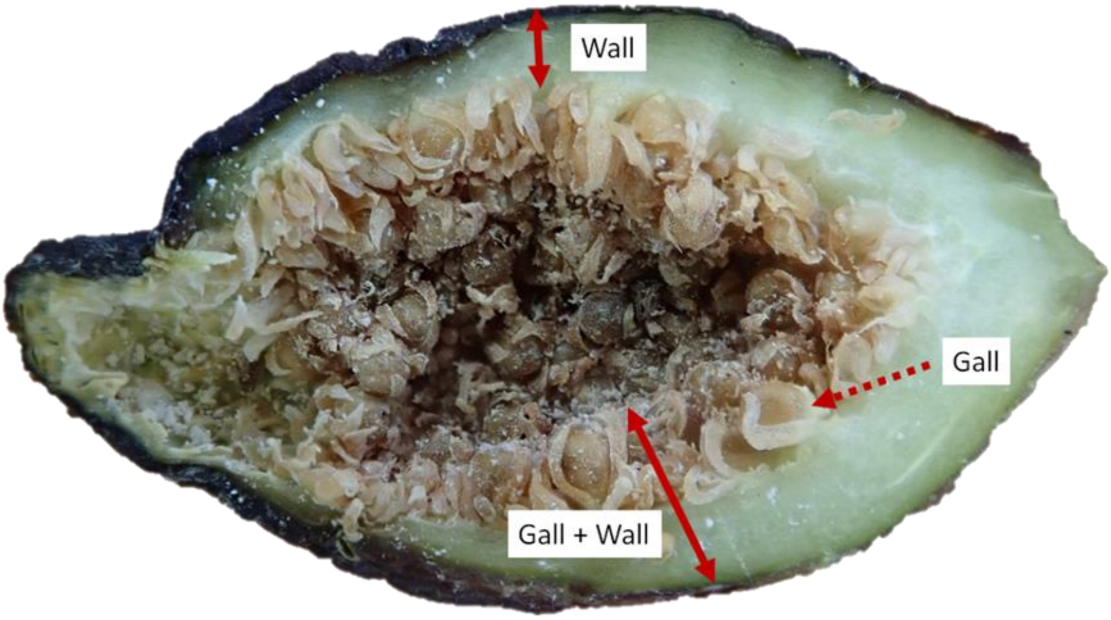

Figure S2 Anatomy of male *Ficus pedunculosa* var. *mearnsii* fig. Note the male flowers had been removed.

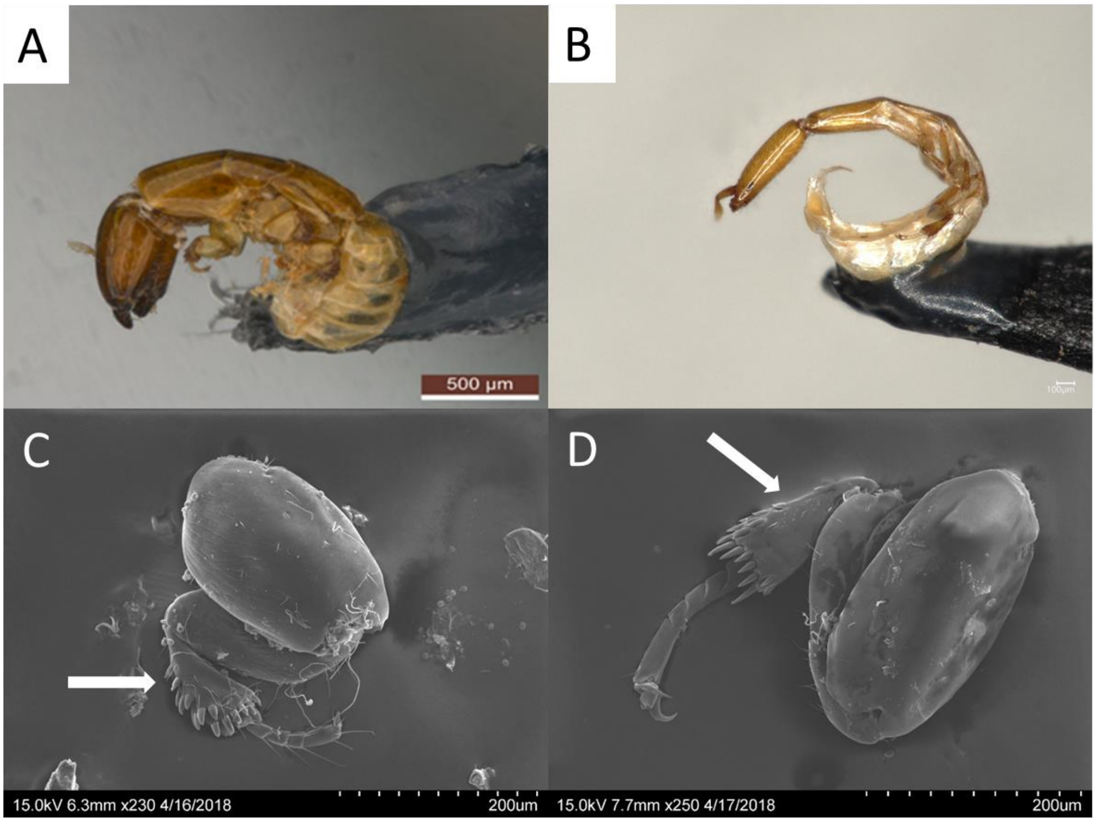

Figure S3 Illustrations of male "*Apocrypta* pedunculosa" associated with *Ficus pedunculosa* var. *mearnsii* and male *A. bakeri* associated with *F. hispida*. (A) Lateral view of "*Apocrypta* pedunculosa". (B) Lateral view of *A. bakeri*. (C) Fore leg of "*Apocrypta* pedunculosa", the arrow pointed more spines on tibia. (D) Fore leg of *A. bakeri*, the arrow pointed fewer spines on tibia.
